# Aβ1-6_A2V_(D) peptide, effective on Aβ aggregation, inhibits tau misfolding and protects the brain after traumatic brain injury

**DOI:** 10.1038/s41380-023-02101-3

**Published:** 2023-05-17

**Authors:** Luisa Diomede, Elisa R. Zanier, Federico Moro, Gloria Vegliante, Laura Colombo, Luca Russo, Alfredo Cagnotto, Carmina Natale, Federica Marta Xodo, Ada De Luigi, Michele Mosconi, Marten Beeg, Marcella Catania, Giacomina Rossi, Fabrizio Tagliavini, Giuseppe Di Fede, Mario Salmona

**Affiliations:** 1 Department of Molecular Biochemistry and Pharmacology, Istituto di Ricerche Farmacologiche Mario Negri IRCCS, Via Mario Negri 2, Milan, Italy; 2 Department of Neuroscience, Istituto di Ricerche Farmacologiche Mario Negri IRCCS, Via Mario Negri 2, Milan, Italy; 3 Neurology V – Neuropathology Unit, Fondazione IRCCS Istituto Neurologico Carlo Besta, Via Celoria 11, Milan, Italy

**Keywords:** Neuroscience, Diseases

## Abstract

Alzheimer’s disease (AD), the leading cause of dementia in older adults, is a double proteinopathy characterized by amyloid-β (Aβ) and tau pathology. Despite enormous efforts that have been spent in the last decades to find effective therapies, late pharmacological interventions along the course of the disease, inaccurate clinical methodologies in the enrollment of patients, and inadequate biomarkers for evaluating drug efficacy have not allowed the development of an effective therapeutic strategy. The approaches followed so far for developing drugs or antibodies focused solely on targeting Aβ or tau protein. This paper explores the potential therapeutic capacity of an all-D-isomer synthetic peptide limited to the first six amino acids of the N-terminal sequence of the A2V-mutated Aβ, Aβ1-6_A2V_(D), that was developed following the observation of a clinical case that provided the background for its development. We first performed an in-depth biochemical characterization documenting the capacity of Aβ1-6_A2V_(D) to interfere with the aggregation and stability of tau protein. To tackle Aβ1-6_A2V_(D) in vivo effects against a neurological decline in genetically predisposed or acquired high AD risk mice, we tested its effects in triple transgenic animals harboring human PS1(M146 V), APP(SW), and MAPT(P301L) transgenes and aged wild-type mice exposed to experimental traumatic brain injury (TBI), a recognized risk factor for AD. We found that Aβ1-6_A2V_(D) treatment in TBI mice improved neurological outcomes and reduced blood markers of axonal damage. Exploiting the *C. elegans* model as a biosensor of amyloidogenic proteins’ toxicity, we observed a rescue of locomotor defects in nematodes exposed to the brain homogenates from TBI mice treated with Aβ1-6_A2V_(D) compared to TBI controls. By this integrated approach, we demonstrate that Aβ1-6_A2V_(D) not only impedes tau aggregation but also favors its degradation by tissue proteases, confirming that this peptide interferes with both Aβ and tau aggregation propensity and proteotoxicity.

## Introduction

Alzheimer’s disease (AD) is a double proteinopathy characterized by amyloid-β (Aβ) and tau pathology and is the most common dementia in older adults [1, 2]. Despite enormous efforts that have been made in the last few decades to find effective therapy, there are still few weapons available to fight this disease. Recently, Lecanemab, a humanized monoclonal antibody that binds with high affinity to Aβ soluble protofibrils, was approved by the US Food and Drug Administration for treating AD patients with mild cognitive impairment or mild dementia [3]. Since the approval of aducanumab [4], this is the second antibody approved for this disease. The benefits associated with using this class of drugs remain to be established. They are very expensive and require continuous monitoring of patients, thus implying a considerable expenditure of resources for the healthcare system. Further, there is still great debate on the ability of these antibodies, particularly aducanumab, to induce small cerebral hemorrhages and amyloid-related imaging abnormalities associated with cerebral edema and neurological disorders [5]. It is therefore clear that AD is still an incurable disease [6]. The reasons for the consistent failure of more than 400 pharmacological trials for AD are unclear and surely not simple to elucidate. Different factors may have contributed to their failures, including late therapeutic interventions along the course of the disease, inaccurate clinical methodologies in the enrollment of patients, and inadequate biomarkers for evaluating drug efficacy [6–11].

One of the major limitations of the approaches used so far in the treatment of AD is the design of putative modifying drugs to target only Aβ, without targeting tau, which is also actively involved in the onset and progression of AD [1, 2]. Although it has long been known that, in the disease condition, tau species can travel from cell to cell, spreading the neurodegenerative pathology, only recently has this protein been considered a druggable target [12, 13]. In the last 15 years, various therapeutic approaches targeting pathological tau forms, mainly tau aggregation inhibitors and anti-tau monoclonal antibodies, have been developed and tested [10]. Some were effective when tested in vitro and in vivo in AD animal models [13]. However, to Aβ targeting compounds, they did not show clinical efficacy. Targeting a single protein is not a winning strategy for fighting a complex disease such as AD. Developing a multitarget therapy capable of acting against both Aβ and tau may represent an innovative pharmacological approach.

We have recently proposed a novel AD bio-inspired strategy stemming from the clinical discovery that the presence of the A2V substitution in the N-terminal domain of Aβ itself plays a protective role in amyloidogenesis by affecting the speed of protein assembly progression and the aggregation kinetics [14–16]. On these bases, we put forward the design of an all-D-isomer synthetic peptide limited to the first six amino acids of the N-terminal sequence of the A2V-mutated Aβ, Aβ1-6_A2V_(D) [17]. This short peptide interacts with full-length Aβ wild-type, interfering with oligomer generation, fibril formation, and amyloid accumulation [18, 19]. It also interferes with Aβ-dependent neurotoxicity and synaptic dysfunction in animal models and reverses the in vitro and in vivo synaptopathy induced by Aβ [16, 20–22]. Moreover, molecular dynamics simulations carried out by our group and other independent scientists showed that the anti-amyloidogenic activity of the Aβ1-6_A2V_(D) peptide is related to its structural flexibility, which facilitates the heterotypic interaction with Aβ, hindering its assembly [15, 23, 24].

We hypothesized that the natural protective action against AD exerted by the A2V substitution is not limited only to the interaction with Aβ but could also involve effects on tau. Accordingly, the in vivo effectiveness against AD of the bio-inspired Aβ1-6_A2V_(D) peptide could be due to additional non-Aβ effects associated with tau-targeting.

To test our working hypothesis, in this study, we first evaluated the ability of the hexapeptide on the aggregation and stability of recombinant tau. To investigate the effect of Aβ1-6_A2V_(D) on tau toxicity, we utilized *Caenorhabditis elegans* as a biosensor [25–27]. This nematode is capable of specifically detecting toxic tau assemblies, particularly oligomeric assemblies, even when administered exogenously, resulting in neuromuscular impairment. By administering brain homogenates from P301L transgenic mice, a well-characterized animal model of tauopathy, to *C. elegans*, we demonstrated a neuronal defect to that observed with oligomeric recombinant tau [27]. This well-validated approach provides a useful tool for screening potential pharmacological agents [27]. To test the relevance of Aβ1-6_A2V_(D) treatment to AD and other dementias involving tau pathology, we used our established traumatic brain injury (TBI) preclinical model [28, 29]. In humans, TBI precipitates tau pathology with an isoform profile and a phosphorylation state similar to AD [30]. We previously showed that, as in AD and other tauopathies, abnormal forms of tau with prion-like properties are generated in TBI and contribute to late neurodegeneration and cognitive decline [28]. We also showed that exposure of worms to brain homogenates from TBI mice induced progressive locomotor dysfunction with neuromuscular damage and impairment of postsynaptic neurotransmission [26]. Importantly, the administration of anti-tau antibodies rescued motor deficits [26]. This indicates the causal role of misfolded/aggregated TBI-generated tau in toxicity and supports the nematode and our TBI mouse models for the assessment of the Aβ1-6_A2V_(D) as a novel therapeutic agent in acquired forms of AD.

Increased risk of many neurodegenerative diseases, including AD, has long been recognized following exposure to TBI, with an estimated 5–10% of dementia in the community thought to be TBI-associated [31–33]. As such, TBI is a leading risk factor for neurodegenerative diseases. Importantly, neurodegeneration following TBI presents a singular opportunity to study the time course and drivers of dementia pathology from a definite point in time. We believe our approach will provide information on the therapeutic properties of Aβ1-6_A2V_(D) not only for AD but also for other *tauopathies* such as TBI-induced neurodegeneration and associated dementia.

## Materials and methods

### Peptide synthesis

Aβ1-6_A2V_(D) (DVEFRH), TAT48-56(D) (GRKKRRQRRR), and Aβ1-6_A2V_(D)-TAT (DVEFRH-GGGG-GRKKRRQRRR) were synthesized by solid-phase peptide synthesis on an automated Alstra synthesizer (Biotage, Uppsala, Sweden) at 0.1 mM scale with NOVASYN-TGA resin (Novabiochem, Darmstadt, Germany) using Fmoc-protected D-amino acids (Sigma Aldrich, Laufelfingen, CH) [16]. Amino acids were activated by a reaction with O-(Benzotriazole-1-yl)-N, N, N’, N’-tetramethyluronium tetrafluoroborate, and N, N-diisopropylethylamine. A capping step with acetic anhydride was included after the last coupling cycle of each amino acid. The peptide was cleaved from the resin with trifluoroacetic acid/thioanisole/water/ phenol/ethanedithiol (82.5:5:5:5:2.5 vol/vol), precipitated, and washed with diethyl ether. The peptides were purified by reverse-phase high-performance liquid chromatography on a semi-preparative C18 column (Waters Corporation, Milford, MA), and their identity was confirmed with a MALDI-TOF spectrometer (Applied Biosystems, Concord, Ontario, Canada). The samples were then freeze-dried and stored at −20 °C until use. The peptide purity was higher than 95%.

### Mice

Male JNPL3 mice expressing 0N4R human tau with the P301L mutation (P301L) were obtained from Taconic Biosciences (New York, USA) (Tau Model 2508). Controls were non-transgenic (Non-tg) male mice with the same mixed C57BL/6, DBA/2, SW genetic background as P301L mice. Triple transgenic mice harboring human PS1(M146 V), APP(SW), and MAPT(P301L) transgenes, referred to as 3xTg-AD, were kindly provided by Dr. Oddo [34]. Male and female C57BL/6 J mice, referred to as wild type (WT), were purchased from Envigo (Italy).

Mice were housed in a specific pathogen-free animal room at a constant temperature of 21 ± 1 °C, humidity 60 ± 5%, with a 12 h light/dark cycle, and ad libitum access to food and water.

Procedures involving animals and their care were conducted in conformity with the institutional guidelines at the Istituto di Ricerche Farmacologiche Mario Negri IRCCS in compliance with national (D.lgs 26/2014; Authorization n. 19/2008-A issued March 6, 2008, by Ministry of Health); Mario Negri Institutional Regulations and Policies providing internal authorization for persons conducting animal experiments (Quality Management System Certificate – UNI EN ISO 9001:2015 – Reg. N° 6121); the NIH Guide for the Care and Use of Laboratory Animals (2011 edition) and EU directives and guidelines (EEC Council Directive 2010/63/UE). The animal facilities meet international standards and are regularly checked by a certified veterinarian who is responsible for health monitoring, animal welfare supervision, experimental protocols, and the review of procedures.

All animal experiments were designed by the ARRIVE guidelines [35], with a commitment to refinement, reduction, and replacement, minimizing the number of mice, while using biostatistics to optimize mouse numbers as in our previous work using the mouse TBI model [28, 36, 37]. Thus, for statistical validity, we used 8 to 10 mice for the behavioral tests and 6 to 10 mice for biomarker and biochemical analysis.

### Traumatic brain injury

Briefly, the controlled cortical impact was induced over the left parietotemporal cortex of 2-month-old 3xTg-AD mice or aged (24-month-old) WT mice. Mice were anesthetized by isoflurane inhalation (induction 3%; maintenance 1.5%) in an N_2_O/O_2_ (70%/30%) mixture and placed in a stereotaxic frame. Mice were then subjected to craniectomy, followed by the induction of controlled cortical impact brain injury, as previously described [36]. Briefly, the injury was induced using a 3-mm rigid impactor driven by an electromagnetically controlled impact device (Impact One^TM^; Leica, Buffalo Grove, IL, USA) rigidly mounted at an angle of 20° from the vertical plane and applied to the exposed dura mater, between bregma and lambda, over the left parietotemporal cortex (anterior-posteriority: −2.5 mm, laterality: −2.5 mm), at an impactor velocity of 5 m/s and deformation depth 2 mm, resulting in a severe level of injury [38]. The craniotomy was then covered with cranioplasty, and the scalp was sutured. Sham age-matched mice received identical anesthesia and surgery without brain injury. Naive mice (at 2 months of age) were not subjected to any experimental procedure.

### Treatment protocol and samples processing

The Aβ1-6_A2V_(D) was administered to 3xTg-AD and 24-month-old WT TBI underwent mice by repeated intranasal administration as described in [39]. In brief, mice were acclimatized for 2 weeks before the surgery and the treatment. Aβ1-6_A2V_(D) was administered to mice at 50 mg/kg body weight (b.w.) (about 5 µL/nostril/mouse, 10 µL total/each treatment session/mouse) starting at 10 min post-TBI and every 48 h up to 6 days post-injury, using a 20 µL pipette and gel loading pipette tip. At each treatment session, the mice were held in place for 30 seconds after the first 5 µL administration and then left free to move in a cage for 30 seconds before administering the remaining treatment/vehicle [39]. Weight was assessed before each intranasal administration. Neurological functions were monitored longitudinally and plasma levels of neurofilament light (NfL) were assessed at sacrifice as detailed in the Supplementary information (sections ‘Behavioural test’ and ‘Plasmatic neurofilament light’). Seven days after surgery, the mice were sacrificed, the brain was removed, and ipsilateral cortical areas were dissected out, immediately frozen on dry ice, and stored at −80 °C until their use for biochemical and *C. elegans* studies. At 4 h, 24 h, 48 h, and 72 h after surgery, a subset of mice (*n* = 3) was sacrificed, and their brains were removed, frozen immediately in liquid nitrogen, and stored at −80 °C until matrix-assisted laser desorption/ionization (MALDI)-imaging analysis. The investigators were blinded to treatment allocation during the assessment of the results.

### *C. elegans* studies

Bristol N2 nematodes were obtained from the *Caenorhabditis elegans* Genetic Center (CGC, University of Minnesota, Minneapolis, MN, USA) and propagated at 20 °C on solid Nematode Growth Medium (NGM) seeded with *E. coli* OP50 (CGC) for food. We used the bleaching technique to prepare age-synchronized animals [40]. *C. elegans* at the first larval stage were then transferred to fresh NGM plates and grown at 20 °C. At the L3-L4 larval stage, nematodes were collected with M9 buffer, centrifuged, and washed twice with 10 mM PBS (pH 7.4) to eliminate bacteria. Worms were incubated for 2 h at room temperature with orbital shaking, in the absence of *E. coli*, with brain homogenates from Non-tg and P301L mice (30 µg protein/100 worms/100 µL 10 mM PBS, pH 7.4) in the presence or absence of 50 µM Aβ1-6_A2V_(D), 50 µM TAT, or 0.1–200 µM Aβ1-6_A2V_-TAT(D). Control worms were treated with 10 mM PBS, pH 7.4 (Vehicle), 50 µM Aβ1-6_A2V_ (D), 50 µM Aβ1-6_A2V_-TAT(D) or 50 µM TAT (100 worms/100 µL). Pericontusional tissue homogenates from young WT TBI mice 12 months post-injury or aged-matched sham mice were administered to worms (30 µg protein/100 worms/100 µL) in the absence or presence of 50 µM Aβ1-6_A2V_-TAT(D). Pericontusional tissue homogenates from 3xTg-AD TBI mice, as well as pericontusional tissues from aged WT TBI, previously treated or not with intranasal 50 mg/kg b.w. Aβ1-6A2V(D), and sacrificed 7 days post-injury (time by which both 3xTg-AD and aged TBI mice showed cognitive impairment), were administered to worms (30 µg protein/100 worms/100 µL 10 mM PBS, pH 7.4). Homogenates from naive or sham mice were administered to worms as controls for 3xTg-AD and aged WT TBI mice, respectively ((30 µg protein/100 worms/100 µL 10 mM PBS, pH 7.4). As an additional negative control, 10 mM PBS, pH 7.4 (100 worms/100 µL) was administered to nematodes. Worms were then plated onto NGM plates seeded with OP50 *E. coli*, grown at 20 °C, and transferred every day to new NGM plates seeded with *E. coli* to avoid overlapping generations. The locomotor activity of nematodes was scored 7 days after the treatment in blind conditions and expressed as the body bends/min [26].

### Statistical analysis

*C. elegans* experiments were performed using 100 worms per group and were repeated at least three times based on methods described on http://www.wormbook.org. No randomization was required for *C. elegans* experiments. For efficacy studies, the mice were assigned to the different experimental groups using randomization lists (www.randomizer.org). All evaluations were done blind to the treatment group and sample identity. The data were analyzed using GraphPad Prism 9.0 software (CA, USA) by Student’s *t*-test, one-way or two-way ANOVA, and Bonferroni’s or Tukey’s *post hoc* test. IC_50_ values were determined using the same software. A *p*-value < 0.05 was considered significant. Data were analyzed by two-way ANOVA for repeated measures in the case of longitudinal assessments (SNAP test), followed by a Sidak *post hoc* test.

## RESULTS

### Effect of the peptide on tau aggregation and toxicity

**Fig. 1:**
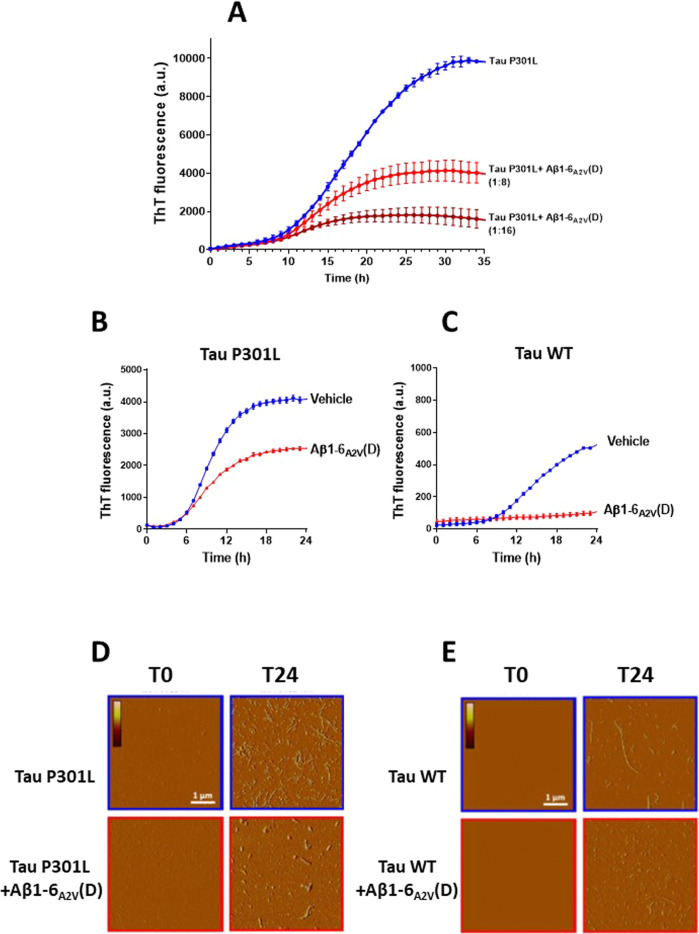
Aβ1-6_A2V_(D) inhibited the aggregation propensity of tau. **A** The aggregation propensity of 10 µM P301L tau was evaluated by fluorometric ThT assay in 10 mM phosphate buffer (PB), pH 7.4, containing a heparin (tau/heparin ratio of 4:1 (w/w)) and 5 mM dithiothreitol (DTT). The ThT fluorescence was measured during incubation at 37 °C in the absence (Tau P301L) and presence of Aβ1-6_A2V_(D) at 1:8 or 1:16 tau-to-peptide molar ratio. Each value represents the mean ± SE from three different experiments (*N* = 3) and is expressed in arbitrary units (a.u.). The aggregation propensity of 2 µM (**B**) Tau P301L and (**C**) Tau WT was evaluated by ThT assay by measuring the fluorescence intensity during incubation at 37 °C in the presence of either 16 µM Aβ1-6_A2V_(D) or the same volume of PB, pH 7.4 (Vehicle). Each value represents the mean ± SE from 3 different experiments (*N* = 4) and is expressed in arbitrary units (a.u.). **D**, **E** Representative atomic force microscopy images were obtained on tau samples immediately before the start of incubation (T0) and 24 h later (T24) the incubation with the peptide under the same experimental conditions described in **B** and **C**. Images were obtained in tapping mode and are shown as amplitude data. Scale bar = 1 µm, color scale: 0–150 mV.

To explore the multitarget potential of the Aβ1-6_A2V_(D) peptide, we conducted a series of studies aimed at evaluating its capacity to counteract tau aggregation and toxicity. We utilized recombinant tau produced in *E. coli*, which lacks post-translational modifications that are typically present in human tau. The kinetics of tau aggregation was monitored using the thioflavin-T (ThT) fluorescence assay and employing heparin as a co-aggregant [41, 42]. Ten µM recombinant tau in P301L-mutated form (Tau P301L) was incubated at 37 °C with different tau-to-peptide molar ratios (1:4, 1:8, and 1:16), in the presence of heparin. The peptide significantly reduced the aggregation propensity of Tau P301L starting from a 1:8 molar ratio tau: Aβ1-6_A2V_(D), and almost complete inhibition of aggregation was observed at a 1:16 molar ratio, as determined by the ThT analysis (Fig. 1A). The molar ratio of 1:8 tau-to-peptide was then selected as the optimal one for additional studies. ThT analysis indicated that 16 µM Aβ1-6_A2V_(D) significantly reduced the aggregation propensity not only of 2 µM Tau P301L but also of Tau wild-type (WT) (Fig. 1B, C). To confirm these results, both recombinant tau isoforms were subjected to atomic force microscopy analysis before (T0) and 24 h (T24) after incubation with the peptide under the same experimental conditions described above (Fig. 1D, E). These data confirm that the peptide inhibits tau aggregation, also in its mutated P301L form, which is known to be more prone to misfold.

**Fig. 2:**
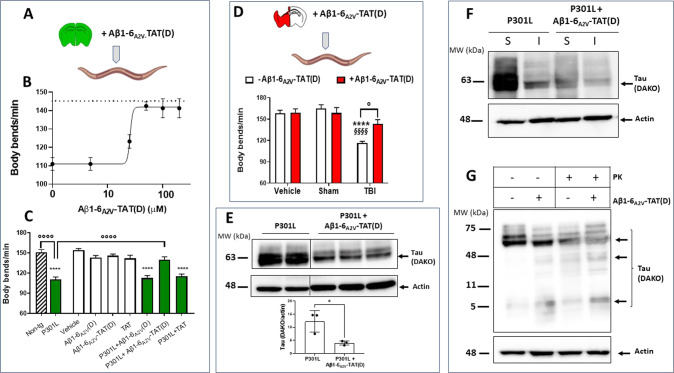
Effect of Aβ1-6_A2V_-TAT(D) on the toxicity caused in *C. elegans* by misfolded tau in P301L and TBI brain homogenates. **A** Brains from P301L mice were homogenized in 10 mM PBS, pH 7.4, and given to worms alone or in the presence of Aβ1-6_A2V_-TAT(D). **B** Worms were administered with brain homogenate (30 µg protein/100 worms/100 µL) in the absence or presence of Aβ1-6_A2V_-TAT(D) (0-200 µM). Control worms were treated with 10 mM PBS, pH 7.4 (100 worms/100 µL) (dotted line). The locomotor activity of nematodes was rated 7 days after treatment. Data are the mean ± SEM of three independent experiments (*N* = 30). **C** Brain homogenates from non-transgenic (Non-tg) and P301L mice were administered to worms (30 µg protein/100 worms/100 µL). Brain homogenate from P301L was also administered in the presence of 50 µM Aβ1-6_A2V_(D), 50 µM Aβ1-6_A2V_-TAT(D), or 50 µM TAT peptide alone. Control worms were treated with 10 mM PBS, pH 7.4 (Vehicle), 50 µM Aβ1-6_A2V_ (D), 50 µM Aβ1-6_A2V_-TAT(D) or 50 µM TAT alone (100 worms/100 µL). The locomotor activity of nematodes was rated 7 days after treatment. °°°°*p* < 0.0001 and ****p < 0.0001 *vs* Vehicle, one-way ANOVA, and Bonferroni’s *post hoc* test. Interaction P301L/ Aβ1-6_A2V_-TAT(D) = 0.001, two-way ANOVA, and Bonferroni’s *post hoc* test. **D** Pericontusional tissues from WT Sham and TBI mice 12 months post-injury (TBI) were homogenized in 10 mM PBS, pH 7.4, and administered to worms (30 µg protein/100 worms/100 µL) in the absence or presence of 50 µM Aβ1-6_A2V_-TAT(D). Control worms were treated with 10 mM PBS, pH 7.4 (100 worms/100 µL) (Vehicle). The locomotor activity of nematodes was rated 7 days after treatment. Data are the mean ± SEM of three independent experiments (*N* = 30). ****p < 0.0001 *vs* Vehicle- Aβ1-6_A2V_-TAT(D), ^§§§§^*p* < 0.0001 *vs* Sham- Aβ1-6_A2V_-TAT(D), °*p* < 0.05, one-way ANOVA and Bonferroni’s *post hoc* test. Interaction TBI/ Aβ1-6_A2V_-TAT(D) = 0.05, two-way ANOVA, and Bonferroni’s *post hoc* test. **E**–**G** Lysates were prepared from the brain homogenates of P301L mice. **E**, **F** P301L brain lysate (30 µg protein/100 µL) was incubated for 2 h at 25 °C with 50 µM Aβ1-6_A2V_-TAT(D) (P301L + Aβ1-6_A2V_-TAT(D)) or 10 mM PBS, pH 7.4 (P301L). **E** Representative western blotting showing the total level of tau and tau quantification expressed as the mean volume of the DAKO band immunoreactivity/actin band. Data are mean ± SD (*N* = 3). *p < 0.05, Student’s *t*-test. **F** Representative western blotting showing the detergent insolubility assay of soluble (S) and insoluble (I) fractions probed with anti-tau DAKO antibody or anti-actin antibody. **G** Effect of Aβ1-6_A2V_-TAT(D) on proteolysis. P301L brain homogenates (30 µg protein/100 µL) were incubated for 30 min at 37 °C with 50 µM Aβ1-6_A2V_-TAT(D) or the equivalent volume of 10 mM PBS, pH 7.4, in the presence or absence of 2.5 µg/mL proteinase K (PK). The reaction was then stopped, and samples were analyzed by western blot. Representative western blotting showing the proteolysis of tau probed with anti-tau DAKO antibody or anti-actin antibody.

To investigate the ability of the peptide to counteract tau toxicity in vivo, we took advantage of the use of *C. elegans* as a biosensor able to specifically recognize the toxic forms of tau. Brain homogenates from P301L transgenic mice were used as a source of misfolded toxic tau [26] and were administered to worms alone or in the presence of an increasing concentration of Aβ1-6_A2V_(D) or Aβ1-6_A2V_-TAT(D). This latter peptide was used since we had previously demonstrated that only in the presence of the TAT sequence, Aβ1-6_A2V_(D) can pass through the worm’s membrane, allowing its protective effects against Aβ toxicity [18]. As shown in Fig. 2A–C, Aβ1-6_A2V_-TAT(D), but not Aβ1-6_A2V_(D), protected worms from the toxicity caused by P301L brain homogenates. The effect was dose-dependent, with an IC_50_ value of 25.60 µM. Peptides alone, at a concentration of 50 µM, did not cause any toxic effects in *C. elegans* (Fig. 2C). We also tested the ability of Aβ1-6_A2V_-TAT(D) to counteract the toxicity of an abnormal form of tau generated in WT mice 12 months after TBI. As previously shown, brain homogenates from WT TBI mice, at variance from those of sham, caused a toxic effect in *C. elegans*, which was completely reverted by 50 µM Aβ1-6_A2V_-TAT(D) (Fig. 2D).

Biochemical analysis was then performed to elucidate the mechanisms underlying this protective effect. The incubation of the P301L brain homogenate with Aβ1-6_A2V_-TAT(D) or Aβ1-6_A2V_(D), but not TAT alone, significantly reduced the tau protein level (Fig. 2E and Supplementary Fig. 1) without affecting the fraction of insoluble tau (Fig. 2F and Supplementary Fig. 2). Notably, the peptide increased the susceptibility of tau to degradation by proteases, and this was particularly evident in the presence of proteinase K (Fig. 2G and Supplementary Fig. 3).

### Efficacy of Aβ1-6_A2V_(D) in young TBI AD-prone mice

**Fig. 3:**
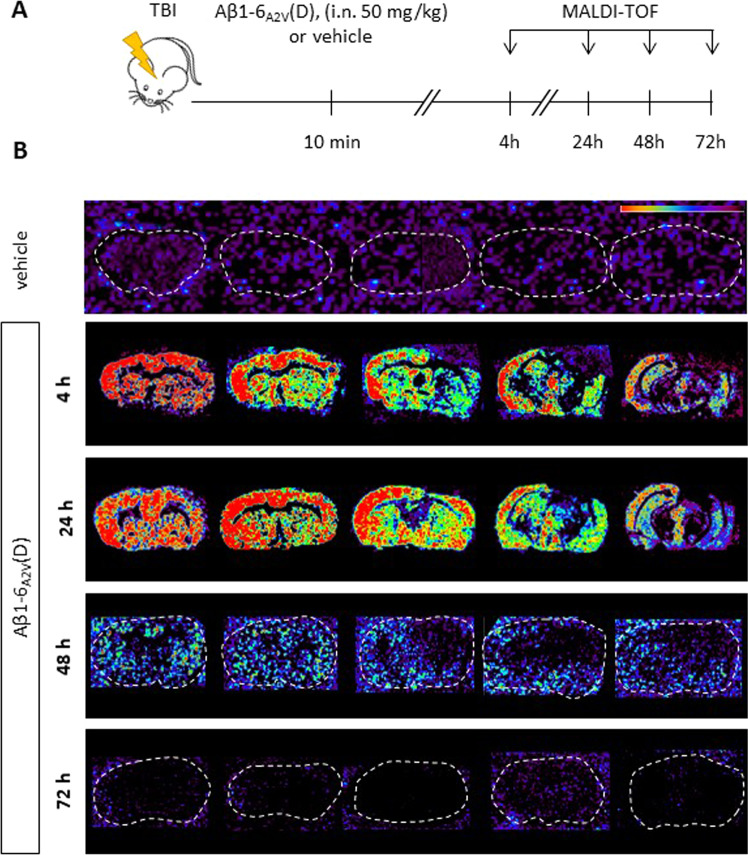
Biodistribution of Aβ1-6_A2V_(D) in brain parenchyma of 3xTg-AD injured mice after intranasal administration. **A** Mice were subjected to TBI, randomly distributed into two groups, and treated intranasally 10 min post-injury with either a single dose of Aβ1-6_A2V_(D) (50 mg/kg b.w.) or saline (vehicle). MALDI-imaging analyses were performed 4-h, 24-h, 48-h, and 72-h post-administration. **B** Representative images of Aβ1-6_A2V_(D) biodistribution assessed by MALDI-TOF and imaged as a heatmap (red > Aβ1-6_A2V_(D) concentration > deep purple).

**Fig. 4:**
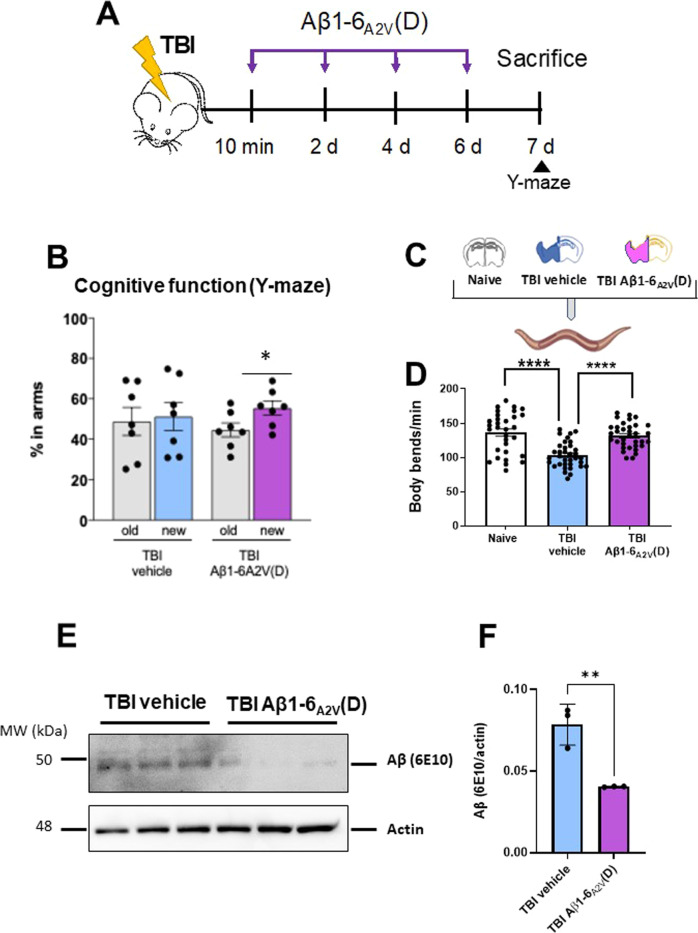
Aβ1-6_A2V_(D) mitigates the cognitive defect of injured 3xTg-AD mice and reverts tau TBI-induced toxicity in *C. elegans*. **A** Schematic representation of the experimental design, Aβ1-6_A2V_(D) treatment paradigm, and cognitive testing. 3xTg-AD TBI mice were administered intranasally with either Aβ1-6_A2V_(D) (50 mg/kg b.w.) or saline (vehicle) 10 min after injury, and then every 48 h thereafter. (**B**) The cognitive function of TBI mice treated with Aβ1-6_A2V_(D) or vehicle was assessed at 7 days by the two-trial Y-maze test. Data are mean ± SEM *p < 0.5 (*n* = 7), paired Student’s *t*-test. **C** Worms were administered with brain homogenate of Naive or 3xTg-AD TBI mice treated with Aβ1-6_A2V_(D) (TBI Aβ1-6_A2V_(D)) or vehicle (TBI vehicle) (30 µg protein/100 worms/100 µL). **D** The locomotor activity of nematodes was rated 7 days after treatment. Data are the mean ± SEM of three independent experiments (*n* = 20). ****p < 0.0001, one-way ANOVA, and Bonferroni’s *post hoc* test. **E** Representative Western blots of Aβ in brain lysates from 3xTg-AD TBI mice treated with vehicle (TBI vehicle) or Aβ1-6_A2V_(D) (TBI Aβ1-6_A2V_(D)). Equal amounts of proteins were loaded in each gel lane and immunoblotted with anti-Aβ (6E10) or anti-actin antibodies. **F** Aβ quantification was expressed as the mean volume of the 6E10 band immunoreactivity/actin band. Data are mean ± SD (*n* = 3). **p < 0.01, one-way ANOVA, and Bonferroni’s *post hoc* test.

TBI accelerates tau deposition in AD-prone mice, especially in 3xTg-AD mice. Therefore, we chose to evaluate the protective effect of Aβ1-6_A2V_(D) in this strain using young TBI mice and applying a treatment schedule that already proved to be effective in a mouse model of AD [16]. We carried out a MALDI-TOF imaging study to assess whether, as observed in APPSwe/PS1dE9 mice [16], the peptide was efficiently distributed in the brain of TBI mice after intranasal administration. Animals that received vehicle (4 h post-TBI) were used as controls. As shown in Fig. 3, 4-h, and 24-h post-injury, the peptide was present in the cerebral cortex, hippocampus, caudate-putamen, and cerebellum. Notably, high Aβ1-6_A2V_(D) levels were detected in the ipsi- and contra-lateral hemispheres that persisted, although at low levels, up to 48 h and were not detectable 72 h after the administration (Fig. 3 and Supplementary Fig. 4-5). Based on these observations and the data already published [16], we selected a treatment schedule consisting of the intranasal administrations of Aβ1-6_A2V_(D) to mice every 48 h. Thus, 3xTg-AD mice were exposed to TBI and treated with 50 mg/kg b.w. Aβ1-6_A2V_(D) or saline starting 10 min after injury, and then every 48 h up to 7 days post-TBI (Fig. 4A). TBI induced a clear spatial memory deficit in the Y-maze at 7 days post-injury, and the Aβ1-6_A2V_(D) administration significantly attenuated the memory impairment, as shown by the increase of the time spent in the new arm of the maze (Fig. 4B).

We previously found that abnormal tau that accumulates in TBI mice is responsible for impairing locomotor function in *C. elegans* [26]. We now asked whether Aβ1-6_A2V_(D) treatment in 3xTg-AD TBI mice was able to reduce the tau and Aβ burden thus mitigating the toxicity of the homogenate. Worms were incubated with brain homogenates from naive mice, and TBI vehicle or Aβ1-6_A2V_(D) treated mice. The body bend frequency was scored after 7 days. Compared to naive homogenates, the TBI vehicle was toxic to *C. elegans* (*p* < 0.001, Fig. 4D) and Aβ1-6_A2V_(D) treatment completely abolished this toxicity (*p* < 0.001 compared to the TBI vehicle). This protective effect was not associated with changes in human tau, P-tau levels as well as P-tau/tau ratio in brain homogenates, possibly explained by the intrinsically high levels of tau P301L expression in this transgenic strain (Supplementary Fig. 6). Notably, Aβ levels were significantly reduced in the brains of the 3xTg-AD TBI mice receiving Aβ1-6_A2V_(D) (Fig. 4E, F) confirming the reduction of amyloid burden in this experimental group. This result may be partially explained by the ability of the peptide to dose-dependently inhibit the activity of BACE in vitro, with an IC_50_ value of 126 nM (Supplementary Fig. 7A). However, the lack of differences in BACE activity between the brain homogenates of 3xTg-AD TBI mice treated or not treated with the peptide (Supplementary Fig. 7B) suggests that the treatment schedule applied may induce short-term changes in vivo that were no longer observable 24 h after administration. It cannot be excluded that other mechanisms leading to the reduction of the Aβ amount, such as an effect of the peptide on its clearance, may contribute to the reduction of amyloid burden.

### Efficacy of Aβ1-6_A2V_(D) in aged TBI mice

**Fig. 5:**
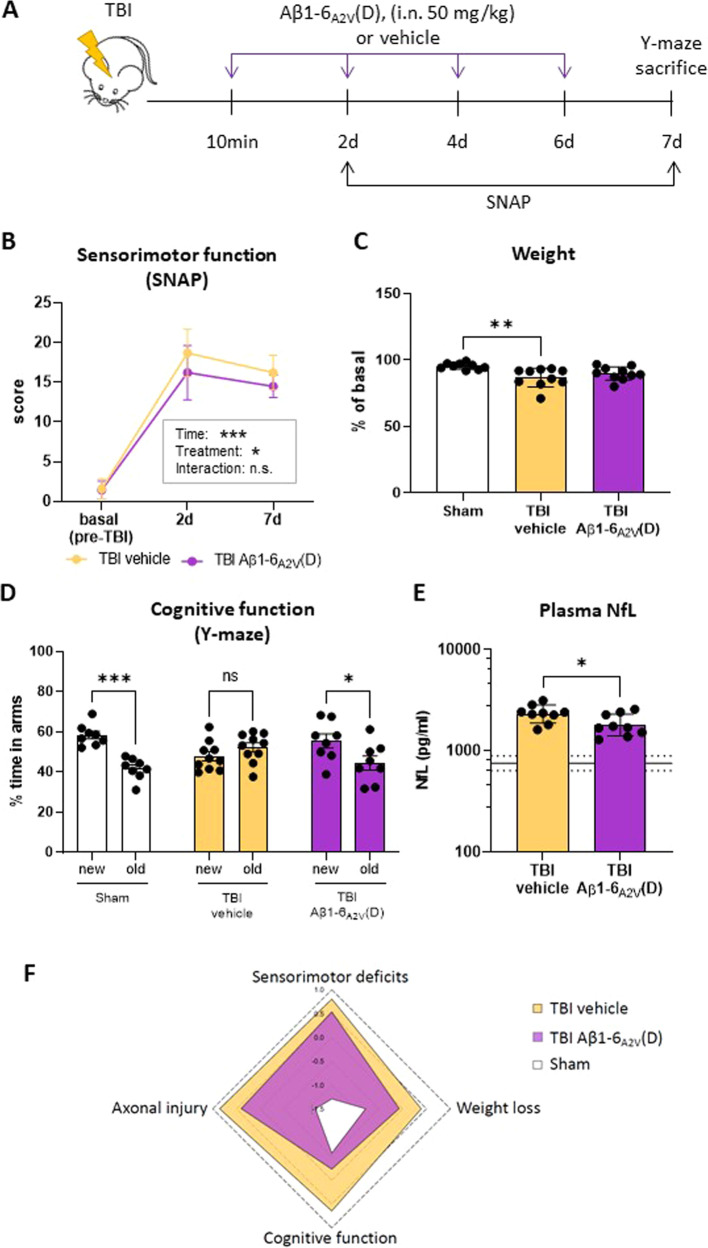
Aβ1-6_A2V_(D) treatment mitigates sensorimotor and cognitive dysfunction and reduces axonal damage in aged WT TBI mice. **A** Schematic of the experimental design, Aβ1-6_A2V_(D) treatment paradigm, and cognitive testing. Aged (24 months of age) TBI WT mice were administered intranasally with 50 mg/kg b.w. Aβ1-6_A2V_(D) (TBI Aβ1-6_A2V_(D)) or saline (TBI vehicle) 10 min after injury, and then every 48 h thereafter. **B** The sensorimotor function was assessed in TBI vehicle- and TBI Aβ1-6_A2V_(D)- treated mice (*n* = 8–10) by SNAP tests 2 and 7 days after injury. The data are mean ± SEM and two-way RM-ANOVA. Significant group effects of time, treatment, and interaction are shown in the box. **C** Body weight was assessed 7 days post-injury and expressed as a percentage over pre-surgery. Data are mean ± SEM (*n* = 8–10), **p < 0.01, one-way ANOVA, and Tukey’s *post hoc* test. **D** Cognitive function was assessed by the two-trial Y-maze test 7 days post-TBI. Data are mean ± SEM, *p ≤ 0.5, ***p < 0.01, paired Student’s *t*-test. **E** Plasma NfL levels were quantified 7 days post-TBI. The dotted lines represent the mean ± SEM of the sham. Data are mean ± SEM (*N* = 8–10), *p < 0.5, unpaired Student’s *t*-test. **F** Radar chart summarizing the effec*t*s of TBI and Aβ1-6_A2V_(D) treatment on multiple parameters including sensorimotor and cognitive functions, weight, and NfL levels as a surrogate marker of axonal injury, expressed as z-score. Results are represented as the mean value.

**Fig. 6:**
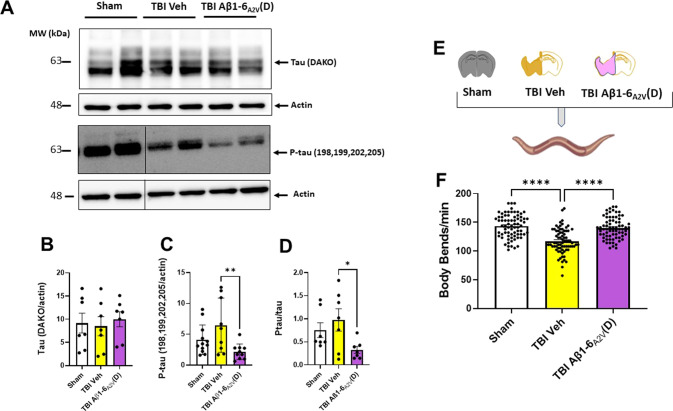
Aβ1-6_A2V_(D) treatment reduces P-tau/tau ratio in the brain of aged WT TBI mice and reverts tau TBI-induced toxicity in *C. elegans*. **A** Representative Western blot of total and phosphorylated (P) tau and the P-tau/tau ratio in lysates from the ipsilateral cortex of aged WT sham and TBI mice treated with either saline (TBI vehicle) or 50 mg/kg b.w. Aβ1-6_A2V_(D) (TBI Aβ1-6_A2V_(D)). Equal amounts of proteins were loaded in each gel lane and immunoblotted with anti-total tau (DAKO), anti-P-tau (198-199-202-205), or anti-actin antibodies. **B**, **C** Quantification of total tau and P-tau assessed in brain lysates. The data are normalized on actin levels and are the mean ± SD (*N* = 6 for tau and *N* = 10 for P.tau). **p < 0.01, one-way ANOVA, and Bonferroni’s *post hoc* test. **D** P-tau/tau was calculated as the ratio of the immunoreactivity signal of P-tau/actin to total tau/actin. Data are mean ± SD (*N* = 7) *p < 0.5, one-way ANOVA, and Bonferroni’s *post hoc* test. **E** Worms were administered with brain homogenate of sham, TBI vehicle, or TBI Aβ1-6_A2V_(D) (30 µg protein/100 worms/100 µL). **F** The locomotor activity of nematodes was rated 7 days after treatment. Data are the mean ± SEM of three independent experiments (*N* = 80). ****p < 0.0001, one-way ANOVA, and Bonferroni’s *post hoc* test.

Similar to humans, normal aging WT mice show an increase in tau deposition in the brain [43–45]. Furthermore, females, compared to male rodents, show increased tau pathology across the parietal–hippocampal network, which is positively correlated with neurological dysfunction [43, 44]. Since in the elderly, women show a higher incidence of TBI due to falls compared to men [46], investigating the effects of Aβ1-6_A2V_(D) in female-aged WT mice may be of high clinical relevance. Furthermore, in contrast to humans, normal aging WT mice do not develop Aβ plaques, likely due to differences in the Aβ sequence between rodents and humans [47, 48]. Thus, experiments in aged female WT TBI mice allow us to test whether Aβ1-6_A2V_(D) protection is maintained in the absence of Aβ pathology (Fig. 5A). TBI Aβ1-6_A2V_(D) treated mice showed a significant improvement in sensorimotor impairments compared to the TBI vehicle up to 7 days after injury (*p* < 0.05, Fig. 5B). By day 7, body weight was decreased in TBI vehicle-treated mice but not in TBI Aβ1-6_A2V_(D)-treated mice, compared to sham mice (Fig. 5C). Similarly, locomotor activity was also reduced in TBI vehicle mice (*p* < 0.05, *vs* sham), while no difference to sham was detected in TBI Aβ1-6_A2V_(D) treated mice (Supplementary Fig. 8). TBI mice showed a clear memory deficit 7 days post-injury in the Y-maze. Aβ1-6_A2V_(D) treatment improved memory function to levels comparable to sham mice (TBI Aβ1-6_A2V_(D): *p* < 0.05, sham: *p* < 0.05 new arm *vs* familiar, Fig. 5D). Moreover, Aβ1-6_A2V_(D) treatment significantly reduced the plasmatic NfL levels (*p* < 0.01 *vs* TBI), indicating the peptide’s ability to protect against the axonal damage caused by TBI [49]. The radar plot in Fig. 5F shows the global effect of Aβ1-6_A2V_(D) on outcome parameters expressed as z-scores. Biochemical analysis performed on the brain homogenates of these mice indicated that the Aβ1-6_A2V_(D) treatment significantly reduced the levels of P-tau and the P-tau/tau ratio (Fig. 6A, C, D), but not the levels of total tau (Fig. 6B). The administration of brain homogenates from the TBI vehicle to *C. elegans* significantly reduced the locomotor activity of the worms after 7 days. This toxic effect was not observed in worms administered with homogenates from Aβ1-6_A2V_(D)-treated mice (*p* < 0.001, Fig. 6E, F).

## Discussion

This study arises from the need to understand more in-depth the protective mechanism of action of Aβ1-6_A2V_(D) against AD pathology that has been previously demonstrated in vitro and in vivo models [16, 22]. With the idea that tau which represents the other relevant key player in the onset and progression of AD, other than Aβ might be a druggable target of the peptide activity we evaluated the potential protective effects of Aβ1-6_A2V_(D) against the toxicity of the tau protein.

TBI is an important environmental risk factor for neurodegenerative diseases such as AD [50–54]. Postmortem brain tissues from patients with a history of TBI display multiple proteinopathies and are common with age-related neurodegenerative diseases [55]. Whether interfering with the proteins that accumulate post-TBI, including tau and Aβ, may confer protection toward the development of dementia later in life and attenuate cognitive dysfunction still needs to be fully addressed.

In this study, we first showed the multitarget potential of Aβ1-6_A2V_(D) by confirming that the peptide inhibited the in vitro aggregation of tau even in its mutated P301L form, known to be more prone to misfold. Next, employing *C. elegans* as a biosensor, we investigated the effect of Aβ1-6_A2V_(D) on the proteotoxicity of tau isoforms [26, 27]. This approach produced remarkable synergies, enabling us to have a deeper insight into the activity of the Aβ1-6_A2V_(D). In particular, the *C. elegans* model provided detailed information at the biochemical level, which is difficult to achieve in vertebrate animals, especially in a short time.

The most relevant observation of our biochemical studies was that the protective effect of Aβ1-6_A2V_(D) can be ascribed to its ability to not only impede tau aggregation but also favor its degradation by tissue proteases. This latter unexpected observation unveils a novel intrinsic mechanism exploited by the peptide that has yet to be clarified.

Lastly, in the TBI model, we tested the relevance of Aβ1-6_A2V_(D) acute treatment to improve neurological outcomes in the context of genetic- or age-related predisposition to AD. TBI by controlled cortical impact leads to the accumulation of hyperphosphorylated tau within 24 h after injury in triple transgenic mice expressing human tau protein and human Aβ peptide [34, 56, 57]. Thus, we first chose 3xTg-AD mice to investigate the protective effect of Aβ1-6_A2V_(D) on TBI. It has recently been shown that a single intranasal administration of the peptide is distributed in the AD mouse brain [16]. Our results confirm and expand these findings by showing that after TBI, Aβ1-6_A2V_(D) can efficiently reach brain areas close to the contusion site with high levels of peptide still detectable 24 h after the administration.

We found that the administration of Aβ1-6_A2V_(D) 10 min after the injury and repeated three times every 48 h prevented the occurrence of memory deficit, a known pathological feature of both 3xTg-AD [58, 59] and TBI [60] mice. Biochemical analysis of the brain homogenates showed a clear reduction of Aβ in mice treated with the peptide, as a result of its multiple mechanisms, including the inhibitory effect of BACE. A similar effect on the processing of Amyloid Precursor Protein (APP) was claimed in subjects carrying the protective A673T substitution in the APP gene [61]. These findings suggest that A2X substitutions in the Aβ sequence may result in multitarget beneficial effects.

Although treatment of TBI 3xTg-AD mice with the peptide did not result in a significant effect on total tau or P-tau levels, it was able to reduce the toxicity of the brain homogenates after administration to the worms proving its capacity in reducing the proteotoxic tau forms. This may depend on different factors, the fact that despite these mice overexpress comparable levels of APP and tau transgenes, Aβ pathology emerges first, then affects the development of tau neuropathology [34].

Next, we tested whether in aged WT mice showing an increased tau deposition in the brain [62] and exposed to TBI as a second neurodegenerative hit [28], Aβ1-6_A2V_(D) treatment was also effective in attenuating cognitive impairment. We observed that the hexapeptide attenuated TBI-induced sensorimotor deficits. This result is in line with the findings that vehicle-treated, but not Aβ1-6_A2V_(D)-treated mice showed a reduction in body weight and locomotor activity, thus suggesting an overall healthier state of aged TBI mice treated with Aβ1-6_A2V_(D). Similar to what was observed in the 3xTg-AD TBI mice, acute spatial memory impairment was prevented by Aβ1-6_A2V_(D) administration. In line with the positive effects of the peptide on functional outcomes, the plasma levels of NfL, a validated marker of axonal injury known to correlate with long-term outcomes after TBI [49] were reduced by treatment. The effect of Aβ1-6_A2V_(D) was modest on the functional outcome but present on multiple outcome parameters including cognitive outcome, weight loss, and axonal damage highlighting its translational value. Moreover, biochemical analysis of the brain homogenates showed a reduction of the P-tau levels and the P-tau/tau ratio [43, 44, 62].

Notably, in aged WT mice, it was not possible to evaluate Aβ levels since the human and murine APP differ at three amino acid residues within the Aβ peptide sequence [63]. These differences, as well as the different cleavage sites of APP by mouse BACE1 [63], diminish the propensity of aged mice to spontaneously develop Aβ plaques.

We previously found that tau pathology is toxic to *C. elegans*, with a selective defect in locomotor activity induced by exposure to chronic but not acute TBI tissue from young WT mice [26]. Here, we showed that in the case of genetically determined or aged-related P-tau pathology, even acute TBI cerebral tissue is toxic to *C. elegans*. In 3xTg-AD mice, Aβ misfolding next to P-tau may also be involved in the observed toxicity. Aβ administration to *C. elegans* induced only pharyngeal impairment, without affecting the motility of worms [18, 20]. In nematodes fed TBI tissues from 3xTg-AD mice, no pharyngeal dysfunction was detected (data not shown), pointing to the primary role of tau in TBI-related toxicity. Importantly, our biosensor approach showed that Aβ1-6_A2V_(D) treatment in both 3xTg-AD and WT-aged TBI mice completely abolished TBI-induced toxicity in the *C. elegans*. Thus, the data highlight a multitarget action of Aβ1-6_A2V_(D) treatment that possibly mitigates both Aβ and tau-induced toxicity after TBI.

Even though supportive treatment for the management of TBI has progressed over the past 20 years, specific neuroprotective strategies are lacking, and there is an urgent need for new therapeutic interventions. Overall, our data show that Aβ1-6_A2V_(D) improved functional outcomes after TBI and reduced the toxicity of proteinopathies triggered by TBI. Importantly, its efficacy in aged TBI mice might highlight its potential application in the elderly population. This could be an important finding, since the number of aged TBI patients is increasing, and age is a strong predictor of mortality and unfavorable outcomes after TBI [64]. Further studies are needed to investigate the efficacy of Aβ1-6_A2V_(D) in relation to age.

Our task cannot be considered complete because Aβ1-6_A2V_(D) might still have other targets driving its protective activity. Additional studies are ongoing to unveil the possible involvement of other mechanisms underlying the protective effect of Aβ1-6_A2V_(D) on the onset and progression of AD as well as other tauopathies.

Finally, we can say that Aβ1-6_A2V_(D) is a multi-target peptide fighting not only against the Aβ toxic pathway but also the tau cascade, as depicted in Supplementary Fig. 9. This ground-breaking observation opens up a world of possibilities, a chance to improve upon the current pharmacological arsenal that only addresses specific targets of AD pathology.

### Supplementary information

Supplemental Information
