# Supplementary material for: Aβ1-6A2V(D) peptide, effective on Aβ aggregation, inhibits tau misfolding and protects the brain after traumatic brain injury
Source: Mol Psychiatry. 2023 May 17;28(6):2433–44. doi: 10.1038/s41380-023-02101-3 (PMC10611578; doi:10.1038/s41380-023-02101-3)
Supplement: Supplementary file 1 — Supplemental Information [file 41380_2023_2101_MOESM1_ESM.docx]

**Supplementary information**

**Aβ1-6_A2V_(D) peptide, effective on Aβ aggregation, inhibits tau misfolding and protects the brain after traumatic brain injury**

Running title: Aβ1-6_A2V_(D) multi-target activity

Luisa Diomede*^1,^**, Elisa R. Zanier*^2,^**, Federico Moro*^2^*, Gloria Vegliante*^2^*, Laura Colombo*^1^*, Luca Russo*^1^*, Alfredo Cagnotto*^1^*, Carmina Natale*^1^*, Federica Marta Xodo*^1^*, Ada De Luigi*^1^*, Michele Mosconi*^1^*, Marten Beeg^1^, Marcella Catania*^3^*, Giacomina Rossi*^3^*, Fabrizio Tagliavini*^3^*, Giuseppe Di Fede*^3^*, Mario Salmona*^1^*

*^1^ Department of Molecular Biochemistry and Pharmacology, Istituto di Ricerche Farmacologiche Mario Negri IRCCS, Via Mario Negri 2, Milan, Italy*

*^2^ Department of Neuroscience Istituto di Ricerche Farmacologiche Mario Negri, IRCCS, Via Mario Negri 2, Milan, Italy*

*^3^* *Neurology V – Neuropathology Unit, Fondazione IRCCS Istituto Neurologico Carlo Besta, Via Celoria 11, Milan, Italy.*

*** These authors contributed equally to this work.

Correspondence to:

Luisa Diomede [luisa.diomede@marionegri.it](mailto:luisa.diomede@marionegri.it)

Mario Salmona [mario.salmona@marionegri.it](mailto:mario.salmona@marionegri.it)

**Recombinant tau**

Recombinant tau wild type (Tau WT) and P301L (Tau P301L) were expressed in *Escherichia coli* and purified by exchange chromatography, followed by size exclusion chromatography [1]. The kinetics of tau aggregation was monitored using the Thioflavin-T (ThT) fluorescence assay. To determine the optimal concentration of Aβ1-6_A2V_(D), different tau-to-peptide molar ratios were tested (1:4, 1:8, and 1:16). To this end, 10 µM Tau P301L in 10 mM phosphate buffer (PB), pH 7.4, were added with heparin (tau/heparin ratio of 4:1 (w/w)) and 5 mM dithiothreitol (DTT). Samples were incubated at 37°C in a low-binding microplate 96-well plate (Corning Incorporated Life Sciences, Acton, MA) in the absence or presence of 40, 80, or 160 µM Aβ1-6_A2V_(D) and 20 µM ThT (100 µL solution per well). ThT fluorescence was measured every 2.5 up to 10 min in static mode using an F500 Infinity plate reader (Tecan Italia Srl, Italy) with an excitation filter of 448 nm and an emission filter of 485 nm. The effect of the Aβ1-6_A2V_(D) at 1:8 tau-to-peptide molar ratio on the aggregation of Tau WT and Tau P301L was also investigated. Two µM Tau, in 10 mM PB, pH 7.4, was incubated as described before in the absence and presence of 16 µM Aβ1-6_A2V_(D) and 20 µM ThT (100 µL solution per well).

Atomic force microscopy (AFM) was then used to visualize the morphology of Tau WT and Tau P301L [1]. Tau samples, at 4 μM in 10 mM phosphate buffered saline (PB), pH 7.4, and containing heparin (tau/heparin ratio 4:1 (w/w)) and 5 mM dithiothreitol, were incubated at 37°C in the presence or absence of 16 µM Aβ1-6_A2V_(D). Immediately before (T0) and 24 h after (T24) incubation, 30 μl of each sample was spotted onto freshly cleaved Muscovite mica disks (Assing) and incubated for 5 min. The mica disks were then washed with 10 ml Milli-Q water and dried under a gentle nitrogen flow for 10 min. Measurements were performed using 0.01–0.025 Ohm/cm antimony-doped silicon probes (T: 3.5–4.5 μm, L: 115–135 μm, W: 30–40 μm, k: 20–80 N/m, f0: 323–380 kHz, Bruker AFM probes) on a multimode AFM, with a Nanoscope V system operating in tapping mode, with a scan rate in the 0.5–1.2 Hz range, proportional to the scanned area. For each sample, five distinct regions were scanned. To minimize possible artifacts, freshly cleaved mica and freshly cleaved mica soaked with 50 mM PB were used as controls.

**Behavioral tests**

**Sensorimotor function.** The effect of the peptide treatment on sensorimotor deficits was assessed on aged WT and sham mice by a Simple Neuroassessment of Asymmetric Impairment (SNAP) test at 2 and 7 days post-TBI. Mice were tested as previously described [2, 3] to assess eight test measures: interaction with the handler, grip strength, visual placing, pacing/circling, gait and posture, head tilt, visual field, coordination, and proprioception. Scores ranged from 0 (normal) to 5 (severely impaired) for each test and were summed to give an overall score that ranged from 0 (best) to 40 (worst).

**Locomotor activity.** The effect of peptide administration on the locomotor activity of aged injured WT mice and sham mice was tested 5 days post-TBI, as previously described [4]. Their behavior was video recorded for 5 min (Ethovision XT 15.0, Noldus). The total distance moved (a measure of locomotor activity) was recorded.

**Cognitive function.** TBI-induced spatial recognition memory deficits were assessed in 3xTg-AD and aged WT mice treated with or without the peptide using the Y-maze test at 7 days post-injury. Mice were tested as previously described [4]. Each mouse was placed in one of the other two open arms (“starting arm”) and allowed to visit the two accessible arms of the maze for 5 min. After an inter-trial interval of 1 h, mice were placed in the same “starting arm” as in trial 1, with free access to all three arms for 5 min (trial 2, retrieval phase). During each trial, the number, and the duration of visits to each arm were recorded (Ethovision XT 15.0, Noldus).

**Plasmatic neurofilament light (NfL)**

Levels of NfL were recorded using commercially available single molecule array assay kits (#103400, Quanterix, Billerica, MA, USA) on an SR-X Analyzer (Quanterix, Billerica, MA, USA). Samples were run in duplicate, and experiments were conducted by researchers blinded to the experimental conditions. The assay’s dynamic range for plasma was 0− ~2000 pg/mL and the lower limit of detection was 0.0552 pg/mL (range 0.0152-0.108 pg/mL).

**MALDI imaging studies**

Studies were carried out as described by Catania et al. [5] to confirm that the intranasal administration of Aβ1-6_A2V_(D) to injured mice allows the efficient distribution of the peptide in the brain tissue. Brains from injured 3xTg-AD mice were used to prepare sagittal sections (14 μm thickness) at -20°C in a cryostat. Fourteen µm coronal sections of fresh-frozen brains were cut and serially collected from bregma +0.8 mm to -2.7 mm at 700 µm intervals for MALDI analysis and Cresyl violet staining to pair Aβ1-6_A2V_(D) biodistribution to anatomical structures. Slices were mounted on steel plate MALDI targets (Opti-TOF High-Resolution T.I.S., Applied Biosystem, Concord, Ontario, Canada) using a small paintbrush, and subsequently placed under vacuum at 4°C overnight and stored at -20°C until use. On the day of the experiment, the mounted tissue sections were coated with a matrix solution of α-Cyano-4-hydroxycinnamic acid (15 mg/ml) dissolved in 60% acetonitrile/0.2% trifluoroacetic acid using a glass nebulizer. The plate was dried at room temperature for 10 minutes and was finally inserted into a MALDI-TOF mass spectrometer (4800 MALDI-TOF, Applied Biosystem, Concord, Ontario, Canada). For brain distribution of the Aβ1-6_A2V_(D) peptide, the molecular weight of the peptide was used as an external standard for generating MALDI-TOF brain imaging.

**Brain homogenates**

Nine-month-old Non-tg and P301L mice were beheaded, the brains removed from the skull, rinsed in cold 10 mM PBS, pH 7.4, immediately frozen, and stored at -80°C until use. Brains from Non-tg and P301L mice and brain cortex obtained from injured, sham, and naïve mice obtained as described above were homogenized in 10% w/v 10 mM PBS, pH 7.4, and the protein concentration were quantified with a Pierce Bicinchoninic Acid (BCA) Protein Assay kit (Life Technologies, Italy) according to manufactory instructions.

**Immunochemical analysis**

Lysates from brain homogenates of P301L mice (30 µg protein/100 µL) incubated for 2 h at 25°C with 50 µM Aβ1-6_A2V_-TAT(D), 50 µM Aβ1-6_A2V_(D), 50 µM TAT or 10 mM PBS, pH 7.4, were analyzed by immunoblotting using 10% SDS-PAGE gel and Western blotting analysis. After heating the samples at 95°C for 10 min in a sample buffer containing 5% β-mercaptoethanol (1:1 v/v, Bio-Rad, Milan, Italy), 30 μg of the proteins were loaded in each lane of the gel. Membranes were blocked for 1 h with 10 mM Tris-HCl solution, pH 7.5, containing 100 mM NaCl, 0.1% Tween 20, 5% low-fat dry milk powder, and 2% bovine serum albumin (Blocking solution) and incubated overnight with the anti-human tau rabbit polyclonal antibody (1:1000, DAKO, Glostrup, Denmark), or anti-actin mouse monoclonal antibody (1:5000, Millipore). Anti-mouse IgG peroxidase conjugate (1:10000, Sigma Aldrich, Milan, Italy) or anti-rabbit IgG peroxidase conjugate (1:10000, Sigma Aldrich, Milan, Italy) were used as secondary antibodies.

Lysates from brain homogenates of P301L mice (50 µg protein/100 µL) incubated for 2 h at 25°C with 50 µM Aβ1-6_A2V_-TAT(D), 50 µM Aβ1-6_A2V_(D) or 10 mM PBS, pH 7.4, were analyzed with an adapted detergent insolubility assay [6]. Briefly, lysates (50 µg) were incubated for 20 min at 4 °C in 50 mM Tris-HCl solution, pH 7.5, containing 0.5% Triton X-100, 0.5% NP-40, and 0.5% sodium deoxycholate. Samples were centrifuged at 150× *g* at 4°C for 5 min, the supernatants were collected and centrifuged at 100,000× *g* at 4°C for 50 min. The supernatant (Soluble fraction) and the pellet (Insoluble fraction) were collected and analyzed in 10% SDS-Page gel. The soluble fraction was heated at 95°C for 10 min in 0.5 M Tris-HCl solution, pH 6.8, containing 10% SDS, 12% β-2-mercaptoethanol, and 50% glycerol and 0.001% bromophenol blue, and 30 μg of proteins were loaded in each gel lane. The insoluble fraction was suspended in 1 mL of 10 mM PBS and centrifuged at 50,000× *g* at 4°C for 30 min. The supernatant was collected and heated at 95°C for 10 min in 1 M Tris-HCl solution, pH 6.8, containing 20% SDS, 24% β-2-mercaptoethanol, 50% glycerol, and 0.001% bromophenol blue, and 30 μg of proteins were loaded in each gel lane. At the end of electrophoresis, gels were blotted onto PVDF membrane, blocked with 10 mM Tris-HCl solution, pH 7.5, containing 100 mM NaCl, 0.1% Tween 20, 5% low fat dry milk powder, and 2% bovine serum albumin, and incubated overnight with anti-human tau rabbit polyclonal antibody (1:1000) or anti-actin mouse monoclonal antibody (1:5000). Peroxidase-conjugated anti-mouse and anti-rabbit IgG (1:10,000) were used as secondary antibodies.

Lysates from areas of 3xTg-AD naïve, and TBI mice treated or not with the hexapeptide were heated at 95°C for 10 min in a sample buffer containing 5% β-mercaptoethanol (1:1 v/v, Bio-Rad, Milan, Italy). Samples were analyzed for tau content by immunoblotting using 10% SDS-PAGE gel (15 µg of proteins/lane) and for Aβ content using 15% SDS-PAGE gel (30 µg of proteins/lane). Membranes were blocked as described before and incubated overnight with the anti-human tau, rat monoclonal antibody (1:1000, Wako FUJIFILM Pure U.S.A. Chemical Corporation), anti-phospho-tau (p-tau) antibody anti-tau paired 198, 199, 202, and 205 (1:2000, Abcam), anti-Aβ mouse monoclonal 6E10 antibody (1:1000, Biolegend), or anti-actin mouse monoclonal antibody (1:5000, Millipore). Anti-mouse IgG peroxidase conjugate (1:10000, Sigma Aldrich, Milan, Italy) and anti-rabbit IgG peroxidase conjugate (1:10000, Sigma Aldrich, Milan, Italy) were used as secondary antibodies. The mean volumes of the immunoreactive bands were recorded using Image Lab™ software (Bio-Rad). The data are expressed as the mean of the immunoreactive bands/volume of total actin-dyed proteins in the spot ± SD.

**Proteinase K digestion**

P301L brain homogenates (30 µg protein/100 µL) were incubated for 2 h at 4°C with 50 µM Aβ1-6_A2V_-TAT(D) or the equivalent volume of 10 mM PBS, pH 7.4, before the treatment with 2.5–10 µg/ml of PK. Thirty minutes after incubation at 37°C, the samples were boiled for 10 min in a sample buffer containing 5% β-mercaptoethanol and loaded in 10% SDS-PAGE. At the end of electrophoresis, gels were blotted onto the PVDF membrane, blocked with 10 mM Tris-HCl solution, as described before, and incubated overnight with anti-human tau rabbit polyclonal antibody (DAKO, 1:1000). Peroxidase-conjugated anti-rabbit IgG (1:10000, Sigma Aldrich) was used as the secondary antibody.

**β-secretase enzyme (BACE) activity**

The ability of Aβ1-6_A2V_(D) to inhibit BACE activity was measured *in vitro* as described in the BACE1 Inhibitor Screening Kit ab283408 (Abcam, Cambridge, UK). Increasing concentrations of Aβ1-6_A2V_(D) were employed. Fluorescence was read for 60 min at 37°C at Ex/Em= 345/500 nm using a spectrophotometer (Infinite M200, Tecan, Männedorf, Switzerland). BACE activity was measured in the ipsilateral cortex of 3xTg-AD TBI mice treated with or without the peptide using the β-Secretase Activity Fluorometric Assay Kit MAK237 (Sigma Aldrich, St. Louis, USA). The protein concentration of the samples was analyzed using the BCA assay, as previously described. The samples were diluted to obtain a final concentration of 25 µg proteins/50 µL and then processed according to the kit instructions. Fluorescence was read at Ex/Em = 355/495 nm.


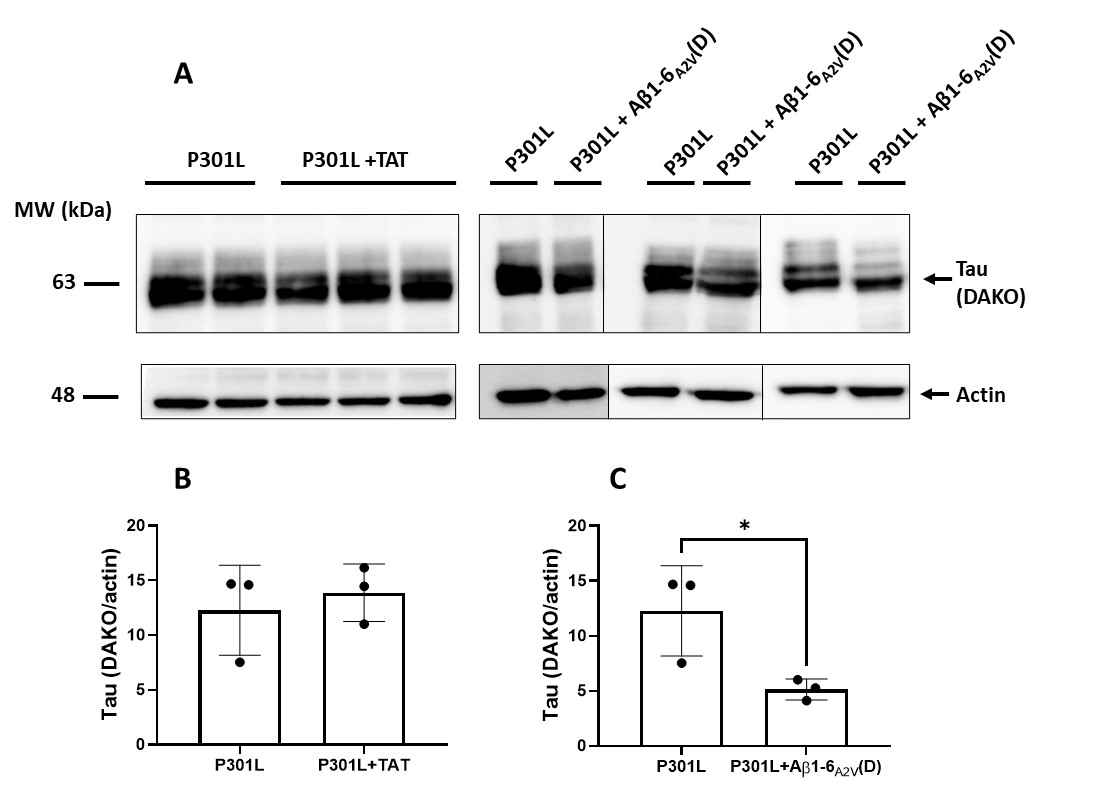


**Supplementary Figure 1. Effect of Aβ1-6_A2V_(D) and TAT on the level of tau in P301L brain homogenates.** (**A**) Representative western blotting showing the total level of tau in lysates prepared from the brain homogenates of P301L mice incubated (30 µg protein/100 µL) for 2 h at 25°C with 50 µM TAT, 50 µM Aβ1-6_A2V_(D) or 10 mM PBS, pH 7.4 (P301L). (**B, C**) Tau quantification is expressed as the mean volume of the DAKO band immunoreactivity/actin band. Data are mean ± SD (N = 3). *p <0.05, Student’s t-test.


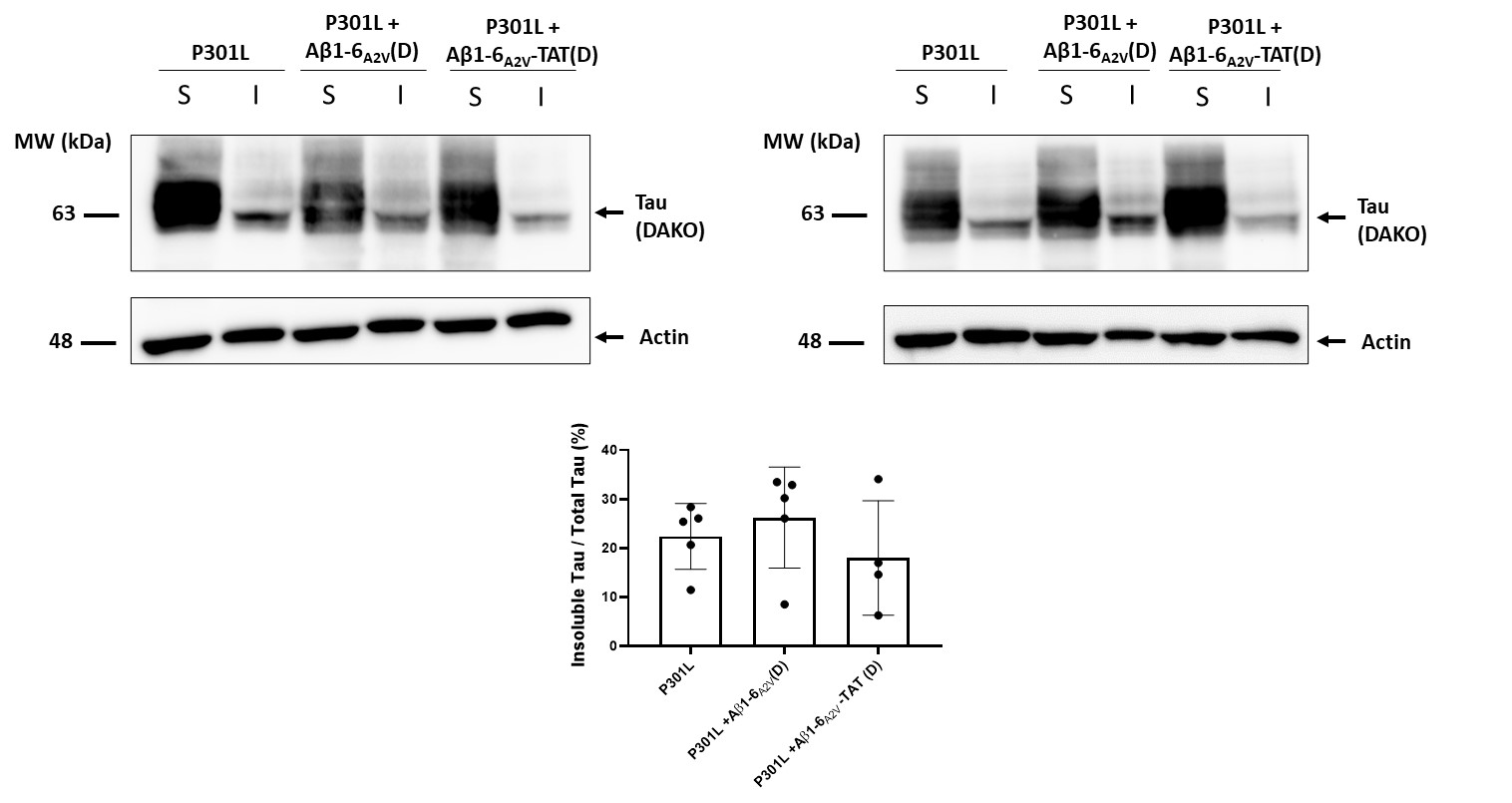


**Supplementary Figure 2. Detergent insolubility assay of brain homogenates from P301L mice incubated with Aβ1-6_A2V_(D) or Aβ1-6_A2V_-TAT-(D).** Representative western blotting of lysates prepared from the brain homogenates of P301L mice incubated (30 µg protein/100 µL) for 2 h at 25°C with 50 µM Aβ1-6_A2V_-TAT-(D), 50 µM Aβ1-6_A2V_(D) or 10 mM PBS, pH 7.4 (P301L), showing the detergent insolubility assay of soluble (S) and insoluble (I) fractions. Tau quantification in the S and I fraction is expressed as the mean percentage immunoreactivity of the DAKO signal in the insoluble fraction/total Tau (soluble + insoluble fraction). Data are mean ± SD (N = 4-5).


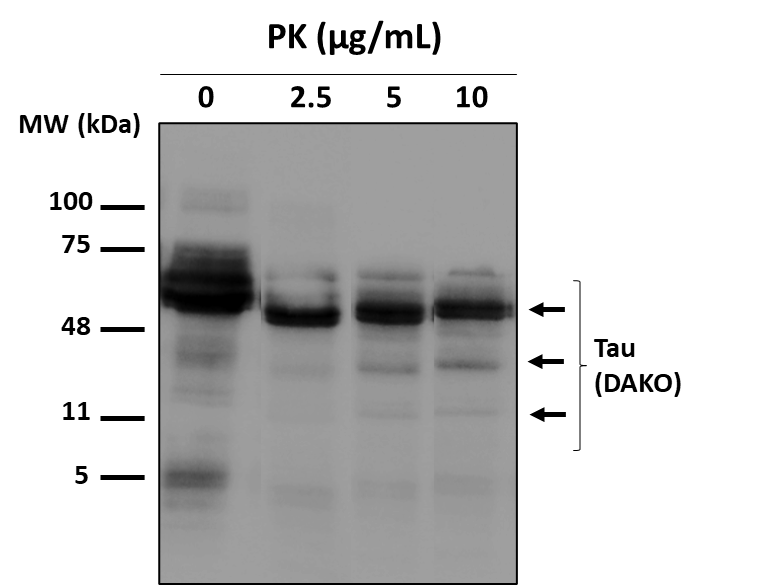


**Supplementary Figure 3.** Effect of 50 µM Aβ1-6_A2V_-TAT(D) on the proteolysis of brain homogenates of P301L mice (30 µg protein/100 µL) caused by incubation for 30 min at 37°C with increasing concentrations of proteinase K (PK). Representative Western blotting showing the proteolysis of tau probed with anti-tau DAKO antibody.


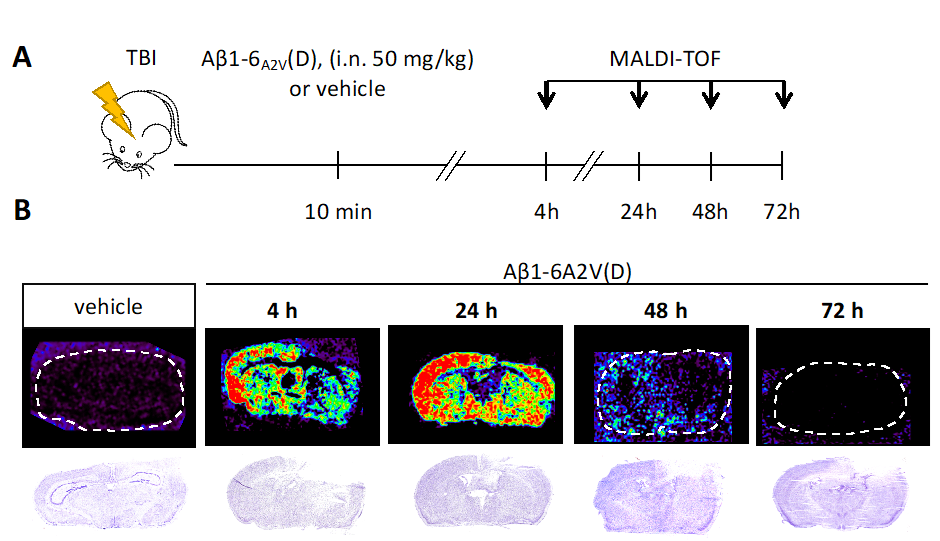


**Supplementary Figure 4**. **Comparison between MALDI imaging and anatomical brain structures.** (A) Mice were subjected to TBI, randomly distributed into two groups, and treated intranasally 10 min post-injury with either a single dose of Aβ1-6_A2V_(D) (50 mg/kg b.w.) or saline (vehicle). MALDI-imaging analyses were performed 4-h, 24-h, 48-h, and 72-h post-administration. (B) Representative images of Aβ1-6_A2V_(D) biodistribution assessed by MALDI-TOF and imaged as a heatmap (red > Aβ1-6_A2V_(D) concentration > deep purple). Cresyl Violet staining was performed to pair Aβ1-6_A2V_(D) biodistribution to anatomical brain structures. A slice of a TBI mouse treated with saline and sacrificed 4-h post-administration was used as control (vehicle).

**
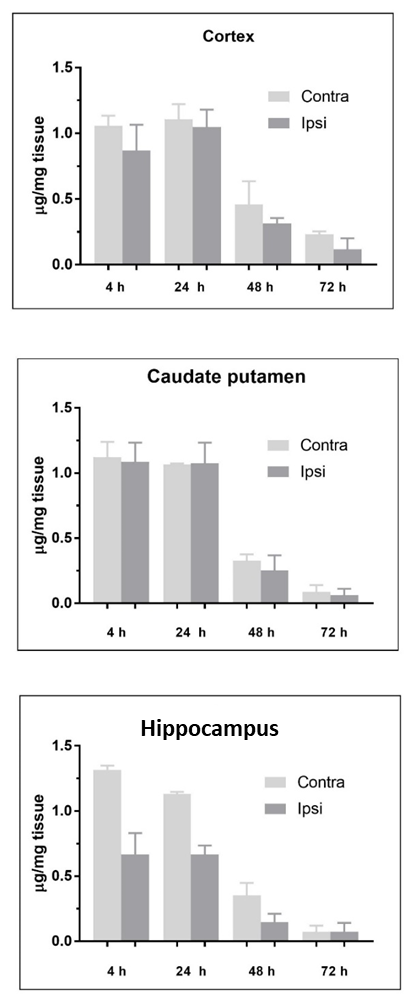
**

**Supplementary Figure 5.** Quantitative distribution of Aβ1-6_A2V_(D) in ipsilateral (Ipsi) traumatic brain injury (TBI) and contralateral (Contra) brain regions of mice administered with the peptide intranasally. Animals were euthanized at 4-, 24-, 48-, and 72 hours post-administration for analysis. Image analysis software was employed to calculate the peptide's relative optical intensity (ROI) in the brain tissues. As previously described, a standard curve was generated using Aβ1-6A2V(D) at concentrations ranging from 0.1 to 2 µg/mg tissue to express the ROI values in µg peptide/mg tissue [5]. The data are represented as mean ± standard deviation (SD) with a sample size of 3-4.


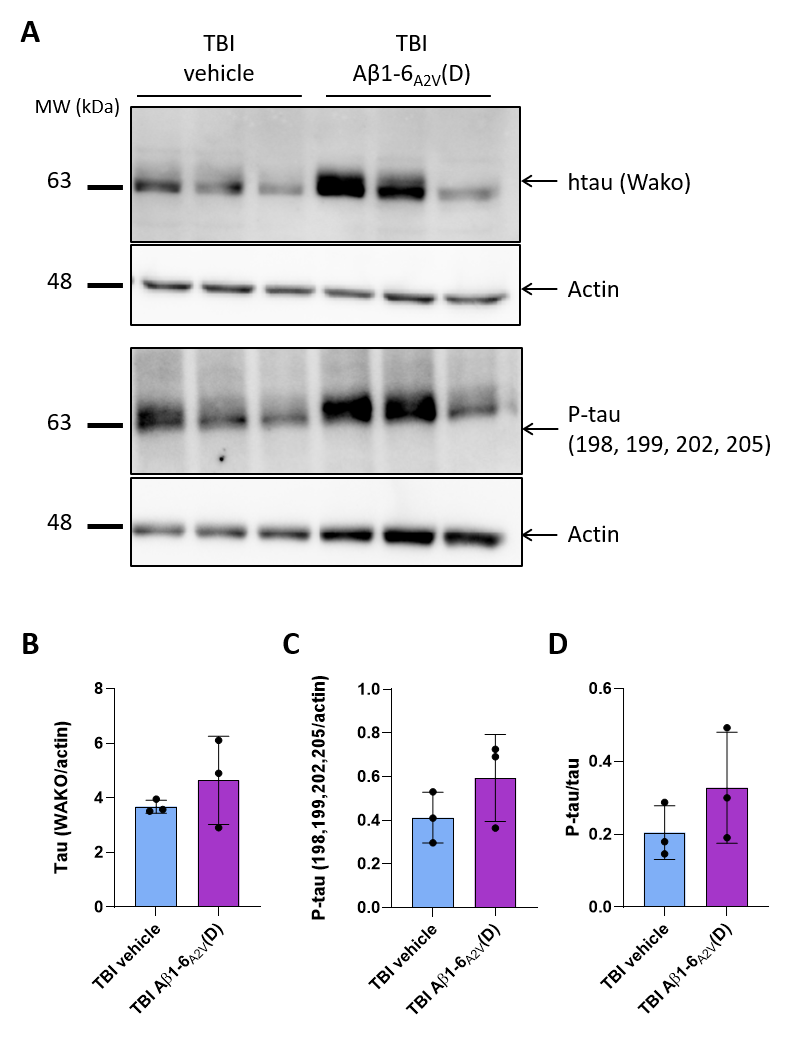


**Supplementary Figure 6. (A)** Representative Western blots of total tau, phosphorylated tau (P-tau), and actin in brain lysates from naive, 3xTg-AD TBI mice treated with vehicle (TBI vehicle), and 3xTg-AD TBI mice treated with 50 mg/kg b.w. Aβ1-6_A2V_(D) (TBI Aβ1-6_A2V_(D)). Equal amounts of proteins were loaded in each gel lane and immunoblotted with anti-human tau (Wako) (htau), anti-P-tau (198-199-202-205), or anti-actin antibodies. (**B, C**) Total tau and P-tau quantification were expressed as the mean volume of the Wako and P-tau band immunoreactivity/actin band. Data are mean ± SD (N= 3). (**D**) The ratio of the immunoreactivity signal of P-tau/actin to Tau Wako/actin. Data are the mean volume of the immunoreactive band/actin ± SD. Data are the mean volume of the immunoreactive band/actin ± SD (N = 3).


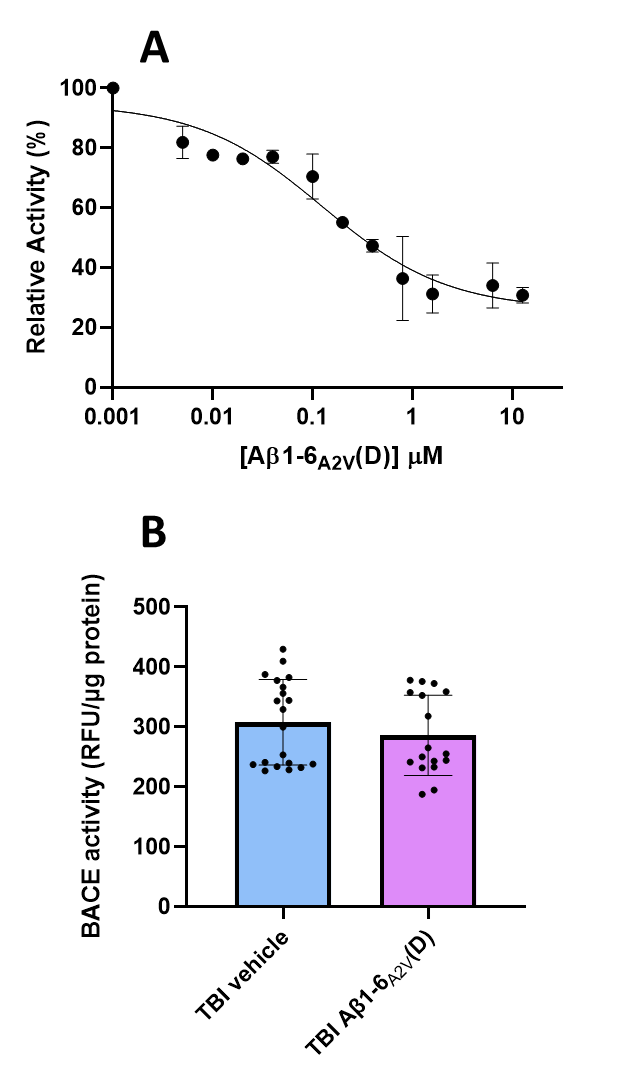


**Supplementary Figure 7. Effect of Aβ1-6_A2V_(D) on the BACE activity.** (**A**) Dose-dependent inhibitory effect of Aβ1-6_A2V_(D) on BACE activity determined *in vitro*. (**B**) BACE activity determined in the brain homogenates of 3xTg-AD TBI mice (N = 3) treated (TBI Aβ1-6_A2V_(D)) or not (TBI vehicle) with 50 mg/kg b.w. Aβ1-6_A2V_(D) and sacrificed 24 h after the last administration. Data are mean ± SD, one-way ANOVA, and Bonferroni’s *post hoc* test.

**
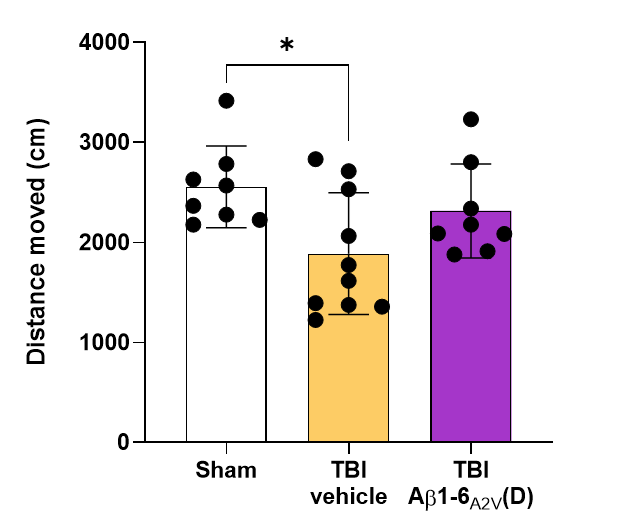
**

**Supplementary Figure 8. Effect of Aβ1-6_A2V_(D) on the locomotor activity of injured WT old mice.** Locomotor activity was evaluated during the open field test 5 days post-TBI in Sham (N = 8), TBI vehicle (N = 10), and TBI Aβ1-6_A2V_(D) (N = 8) mice. Data are mean ± SEM. *p <0.01, one-way ANOVA, and Tukey’s *post hoc* test.


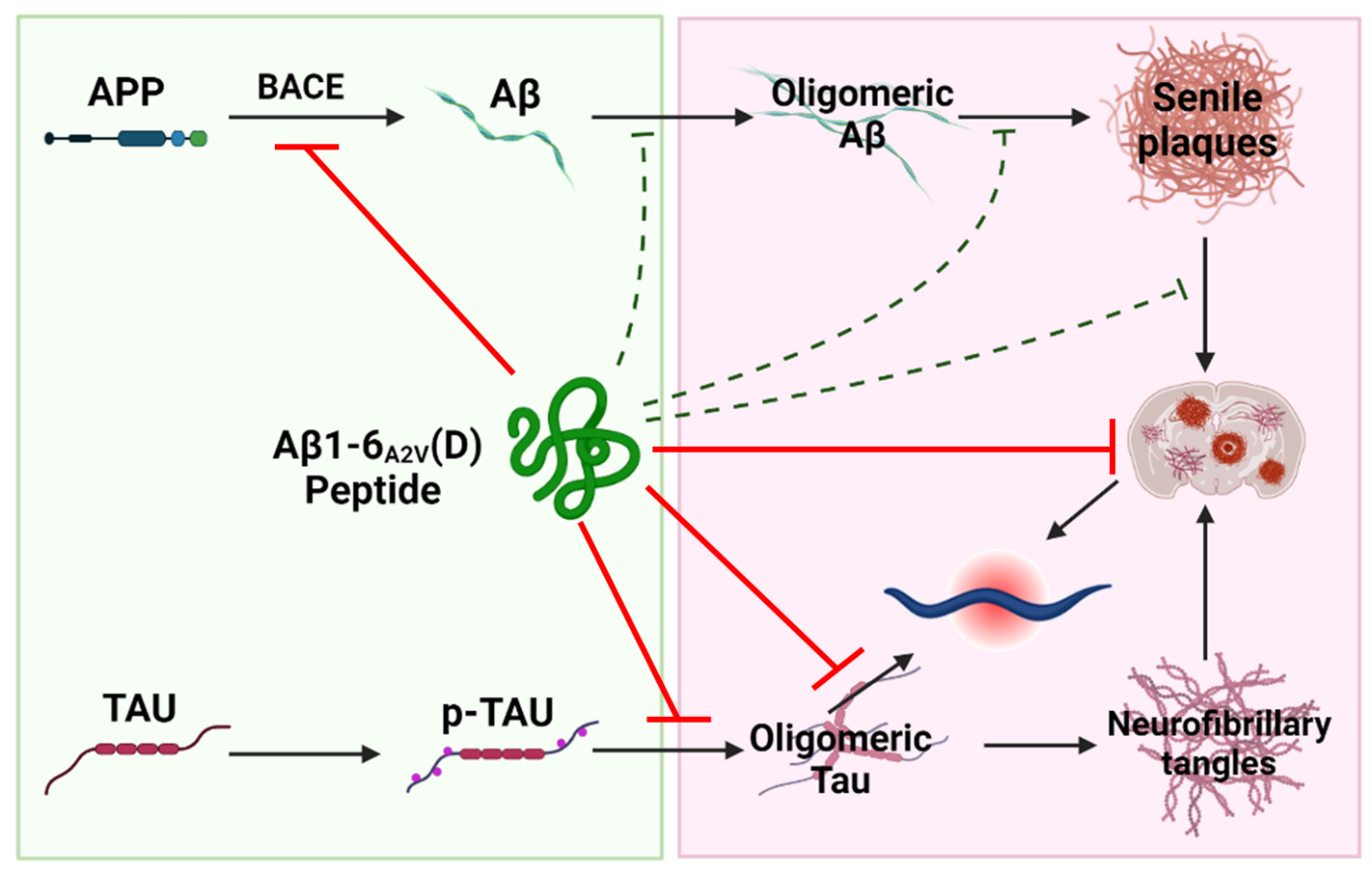


**Supplementary Figure 9. Representation of the multitarget activity of the Aβ1-6_A2V_(D) peptide**. In our previous studies, we reported that the Aβ1-6_A2V_(D) peptide can inhibit the formation of oligomers of the Aβ protein, the formation of Aβ fibrils, and the accumulation of amyloid deposits in animal models (represented by the dashed green lines), ultimately preventing synaptic damage. The results of our current study further support the peptide's multi-target effects, including the inhibition of the BACE enzyme (represented by the solid red line), responsible for cleaving the APP protein and accumulating neurotoxic forms of Aβ. Moreover, the peptide can facilitate tau degradation, counteracting its aggregation and the formation of oligomers that have been recognized as toxic by *C. elegans* (represented by the solid red lines). Notably, we found that treatment with Aβ1-6_A2V_(D) in mice with traumatic brain injury led to improved neurological outcomes and reduced blood markers of axonal damage (represented by the solid red line), providing further evidence of the peptide's potential therapeutic value. Overall, our data strongly support the peptide's ability to act on various interlinked pathways involved in the onset of neurodegeneration.

**REFERENCES**

1. Zanier ER, Barzago MM, Vegliante G, Romeo M, Restelli E, Bertani I, et al. C. elegans detects toxicity of traumatic brain injury generated tau. Neurobiol Dis. 2021;153:105330.

2. Moro F, Pischiutta F, Portet A, Needham EJ, Norton EJ, Ferdinand JR, et al. Aging is associated with maladaptive immune responses and worse outcomes after traumatic brain injury. Brain Communications. 2022;4:fcac036.

3. Moro F, Fossi F, Magliocca A, Pascente R, Sammali E, Baldini F, et al. Efficacy of acute administration of inhaled argon on traumatic brain injury in mice. British Journal of Anaesthesia. 2021;126:256–264.

4. Di Sapia R, Moro F, Montanarella M, Iori V, Micotti E, Tolomeo D, et al. In-depth characterization of a mouse model of post-traumatic epilepsy for biomarker and drug discovery. Acta Neuropathologica Communications. 2021;9:76.

5. Catania M, Colombo L, Sorrentino S, Cagnotto A, Lucchetti J, Barbagallo MC, et al. A novel bio-inspired strategy to prevent amyloidogenesis and synaptic damage in Alzheimer’s disease. Mol Psychiatry. 2022. 26 August 2022. https://doi.org/10.1038/s41380-022-01745-x.

6. Kaufman SK, Sanders DW, Thomas TL, Ruchinskas AJ, Vaquer-Alicea J, Sharma AM, et al. Tau Prion Strains Dictate Patterns of Cell Pathology, Progression Rate, and Regional Vulnerability In Vivo. Neuron. 2016;92:796–812.
